# Antibacterial mechanism of pulsed electric field-assisted extracted volatile oil from *Wurfbainia villosa* leaves against *Escherichia coli* and *Staphylococcus aureus*

**DOI:** 10.3389/fmicb.2026.1828154

**Published:** 2026-05-19

**Authors:** Yuancong Gu, Bangyu Lv, Xinrui Xie, Xingrui Nian, Xinhe Yang

**Affiliations:** 1 College of Food Science and Engineering, Guangdong Ocean University, Yangjiang, China; 2 Yangjiang Research Institute of Guangdong Ocean University, Yangjiang, China

**Keywords:** antibacterial activity, *Escherichia coli*, natural antimicrobials, *Staphylococcus aureus*, volatile oil, *W. villosa*

## Abstract

**Introduction:**

This study employed pulsed electric field-assisted steam distillation (PEF-HD) to optimize the extraction of volatile oil from *Wurfbainia villosa* leaves (WEO).

**Methods:**

Optimization was performed using single-factor experiments and response surface methodology. The extracted oil was analyzed by GC-MS for chemical composition, and its antibacterial mechanism against *Escherichia coli* (*E. coli*) and *Staphylococcus aureus* (*S. aureus*) was assessed.

**Results:**

The optimal conditions were an electric field intensity of 0.64 kV/cm, 284 pulses, and a 1:6 g/mL solid–liquid ratio, yielding 0.779 ± 0.034% oil. This represented a 1.32-fold increase over traditional steam distillation. GC-MS analysis identified 59 components, dominated by terpenes (66.74%) and oxygenated terpenes (25.82%), together comprising 92.56% of the total oil. The oil disrupted cell wall integrity and membrane permeability in both *E. coli* and *S. aureus*, causing protein and nucleic acid leakage and leading to cell death.

**Discussion:**

In summary, PEF-HD improved extraction efficiency and yielded a terpenoid-rich oil that exhibited antibacterial activity against foodborne pathogens by disrupting cell membranes. These findings provide a scientific foundation and technical framework for the development and utilization of *W. villosa* leaf resources as antibacterial agents.

## 1 Introduction

*Wurfbainia villosa* (Lour.) Škorničk. and A.D.Poulsen (*W. villosa*) is a medicinal and edible plant. Native to Yangchun City, Guangdong Province, it is a characteristic authentic medicinal herb and one of the “Four Great Southern Medicinal Herbs.” It possesses a pungent taste and warm, aromatic properties, and is traditionally used to promote qi circulation, regulate the middle jiao, harmonize the stomach, invigorate the spleen, resolve dampness, and disperse stagnation (Gu et al., 2024). All parts of the plant—including its flowers, fruits, leaves, and roots—are used medicinally, leading to its reputation as “Ginseng in the North, Chunsharen in the South” (Lai et al., 2024). Current research has primarily focused on the fruit, while the development and utilization of *W. villosa* leaf resources remain limited. The leaves are abundant but are often discarded or used as animal feed. Previous studies have indicated that the volatile oil components of *W. villosa* leaves are similar to the aromatic constituents of its fruit and exhibit bioactive effects including promoting skin wound healing, anti-inflammatory and analgesic activity, and enhanced gastrointestinal motility (Huang et al., 2017; Wang, 2017). Based on these properties, the volatile oil from *W. villosa* leaves holds potential for functional food applications, particularly as a natural preservative and antimicrobial agent. By inhibiting microbial growth, it can extend the shelf life of food products and be applied to ready-to-eat meals or fruit and vegetable preservation. This aligns with consumer demand for clean-label natural preservatives and offers a new approach for the high-value utilization of *W. villosa* leaf resources.

Volatile oils are important carriers of secondary metabolites and represent a significant source of potential antibacterial compounds (Rosato et al., 2007). Among common pathogenic bacteria, *Staphylococcus aureus* (*S. aureus*) and *Escherichia coli* (*E. coli*) serve as representative Gram-positive and Gram-negative strains, respectively, and are major foodborne pathogens (He J. T. et al., 2025). Existing studies have demonstrated that various plant volatile oils exhibit strong antibacterial activity against these bacteria. For example, *Chrysanthemum* volatile oil shows significant inhibitory effects against both *E. coli* and *S. aureus* (Sakhawy et al., 2025); volatile oils from *P. sylvestris* cones, *P. halepensis* cones, and their needles display notable antimicrobial efficacy (Mirković et al., 2024); and *Tagetes minuta* volatile oil exhibits strong antibacterial activity against both Gram-positive and Gram-negative bacteria, with highly lethal effects within a short period (1 h), including complete eradication of *E. coli* (Bordón et al., 2025). Furthermore, volatile oils from *Vanilla* leaves and buds have been demonstrated to have stronger antibacterial effects against *S. aureus* and *E. coli* than certain antibiotics (Samiah et al., 2023). Systematic studies on the antibacterial activity and mechanism of action of volatile oil from *W. villosa* leaves against foodborne pathogens remain scarce.

Pulsed electric field (PEF) pretreatment is an efficient, green, and non-thermal technology characterized by short duration, high efficiency, and environmental friendliness. PEF induces electroporation by applying short high-voltage pulses between two electrodes (typically at 0.1–40 kV/cm), enhancing cell membrane permeability, improving solvent penetration, and promoting the release of bioactive substances (Decker et al., 2025; Punzalan et al., 2025; Xu et al., 2024). This technology has achieved significant progress in microbial inactivation, enzyme inactivation, agricultural product pretreatment, and material extraction, with applications in practical production (Ranjha et al., 2021). In the extraction of plant volatile oils, polyphenols, polysaccharides, and other components, PEF induces cell membrane permeabilization via high-voltage short pulses (Anwar et al., 2021; del Carmen Razola-Díaz et al., 2024; Ferraz and Silva, 2025). The degree of permeabilization depends on the number and size of pores formed; more intensive treatments create larger pores, thereby promoting higher extraction yields (Negin et al., 2023; Marín-Sánchez et al., 2025). For instance, in the extraction of *eucalyptus* and *rosemary* volatile oils, PEF pretreatment increased yields by 40% compared to traditional methods (Barros et al., 2022). However, the application of PEF technology to extract volatile oil from *W. villosa* leaves remains unreported, underscoring its research value.

This study aimed to optimize pulsed electric field-assisted hydrodistillation (PEF-HD) for the extraction of volatile oil from *W. villosa* leaves, to characterize its chemical composition, and to elucidate its antibacterial mechanism against *E. coli* and *S. aureus*, thereby supporting the development of this plant resource as a source of natural antibacterial agents.

## 2 Materials and methods

### 2.1 Materials and reagents

Plant Material: Fresh leaves of *W. villosa* were collected on October 16, 2024, from a cultivation base in Chenwuzhai, Chuncheng Subdistrict, Yangchun City, Guangdong Province, China, and immediately stored at −20°C until use.

Bacterial strains: *E. coli* (CMCC 44103) and *S. aureus* (CMCC 26003) were obtained from the laboratory collection.

Chemical Reagents: Anhydrous sodium sulfate (analytical grade), sodium chloride (analytical grade), and absolute ethanol (analytical grade) were purchased from Sinopharm Chemical Reagent Co., Ltd. (China). Sodium dihydrogen phosphate and disodium hydrogen phosphate (both analytical grade) were sourced from Guangdong Guanghua Sci-Tech Co., Ltd. (China). Tryptone (biological grade), yeast extract powder (biological grade), nutrient agar (biological grade), ampicillin susceptibility test discs, and blank susceptibility test discs were procured from Hubei Bikeman Holding Co., Ltd. (China). An alkaline phosphatase (AKP) assay kit and propidium iodide (PI, ≥ 95%) were supplied by Shanghai Yuanye Bio-Technology Co., Ltd. (China). Glutaraldehyde fixative (for electron microscopy) was acquired from Shanghai Macklin Biochemical Technology Co., Ltd. (China).

### 2.2 Instruments and equipment

Five milliliter volatile oil extractor; TC3K-H electronic balance (Changshu Shuangjie Testing Instrument Factory, China); ME 204 analytical balance (readability: 0.1 mg; Mettler-Toledo Instrument (Shanghai) Co., Ltd., China); SKM digital constant-temperature electric heating mantle (2000 mL); JYL-C 23 juice extractor and blender (Joyoung Co., Ltd., China); PEF-SY-12 K high-voltage pulsed electric field system (Guangzhou Paihu Technology Co., Ltd., China); Thermo Scientific Apreo 2 S scanning electron microscope (Thermo Fisher Scientific, United States); Agilent 7000 D gas chromatography–mass spectrometer (Agilent Technologies Inc., United States); THZ-82 A water bath constant-temperature shaker (Jintan Shuibei Youlian Instrument Factory, China); SpectraMax iD3 microplate reader (Molecular Devices (Shanghai) Co., Ltd., China); TU-1901 UV-Vis spectrophotometer (Beijing Purkinje General Instrument Co., Ltd., China); FE 38 conductivity meter (Mettler-Toledo Instrument (Shanghai) Co., Ltd., China); LGJ-10 C vacuum freeze dryer (Shanghai Borden Biological Technology Co., Ltd., China); EVOS Auto 2 inverted fluo-rescence microscope (Thermo Fisher Scientific, United States); and SW-CJ-2 FD clean bench (Suzhou Antai Air Technology Co., Ltd., China).

### 2.3 Extraction of volatile oil from *W. villosa* leaves (WEO) by PEF-HD

Fresh *W. villosa* leaves were cut into segments approximately 3 cm long. Exactly 50.0 g of leaf segments were mixed with a specified volume of distilled water. After soaking for 1 h, the mixture was treated in a PEF chamber. The volatile oil was then extracted by hydrodistillation according to General Rule 2204 of the Chinese Pharmacopoeia (2020 Edition). The volatile oil yield was calculated using Equation (1):


$$\text{Y}=\frac{\text{m}}{\text{M}}\times 100\%$$ (1)

Where: Y is the extraction yield of volatile oil (g/100 g); m is the mass of volatile oil (g); M is the dry weight of *W. villosa* leaves (g).

### 2.4 Single-factor experiments

For the single-factor experiments, the levels for each factor were: electric field intensity (0.31, 0.64, 0.98, 1.31, 1.60 kV/cm), pulse number (120, 180, 240, 300, 360), and solid-liquid ratio (1:4, 1:6, 1:8, 1:10, 1:12 g/mL). The individual effects of these factors on the volatile oil yield from *W. villosa* leaves were investigated. The yield was calculated using Equation (1).

### 2.5 Box-Behnken design (BBD)

Based on the single-factor results, electric field intensity, pulse number, and solid-liquid ratio were selected as independent variables, with volatile oil yield as the response. A three-factor, three-level Box-Behnken design (Table 1) was employed for response surface analysis of the volatile oil yield.

**TABLE 1:** Factors and levels for the Box-Behnken design.

Factors	Levels		
	−1	0	1
Electric field intensity (kV/cm)	0.31	0.645	0.98
Pulse number (times)	240	300	360
Solid-liquid ratio (g/mL)	1:4	1:6	1:8

### 2.6 Chemical composition analysis by GC-MS

GC Conditions: Separation was performed on a DB-WAX capillary column. The injector temperature was 250 °C, with helium carrier gas at 1.2 mL/min in splitless mode. The oven temperature program was: 40 °C (hold 3 min), increased to 130 °C at 3 °C/min (hold 3 min), then to 190 °C at 6 °C/min (hold 3 min), and finally to 240 °C at 10 °C/min (hold 3 min).

MS Conditions: The mass spectrometer was operated in electron ionization (EI) mode at 70 eV. The ion source temperature was 230 °C. Data were acquired in full-scan mode over an *m/z* range of 50–500, with a 3 min solvent delay.

### 2.7 Scanning electron microscopy (SEM) analysis

Untreated and PEF-treated *W. villosa* leaf samples were freeze-dried and sputter-coated with gold. The surface microstructure was observed at 2,500 × magnification with an accelerating voltage of 5–8 kV and a working distance of 10–11 mm.

### 2.8 Filter paper disk diffusion assay

Antibacterial activity was assessed using the filter paper disk diffusion method (Zhang et al., 2024). Briefly, 100 μL of bacterial suspension (1 × 10^6^ CFU/mL) was spread evenly on sterile LB agar plates. Sterile filter paper disks (6 mm diameter) were placed on the agar, and 5 μL of WEO was applied to each disk. Ampicillin disks (10 μg/disk) were used as positive controls. After incubation at 37 °C for 24 h, inhibition zone diameters were measured using the vernier caliper. All experiments were performed in triplicate, and results are presented as mean values.

### 2.9 Determination of minimum inhibitory concentration (MIC) and minimum bactericidal concentration (MBC)

The minimum inhibitory concentration (MIC) of WEO against *E. coli* and *S. aureus* was determined using the two-fold dilution method (Wang et al., 2021). Seven sterile tubes, each containing 1 mL of LB broth, were prepared. To the first tube, 1 mL of WEO stock solution (102.4 mg/mL) was added and mixed. A serial twofold dilution was then performed by transferring 1 mL from the first tube to the second, mixing, and repeating sequentially through the seventh tube, discarding 1 mL from the last tube. Subsequently, 1 mL of bacterial suspension (1 × 10^6^ CFU/mL) was added to each tube. A blank control (sterile medium alone) and a growth control (medium with bacterial suspension but without WEO) were included. After incubation at 37 °C with shaking for 24 h, OD_600_ was measured and tube turbidity was recorded. Turbidity with increased OD indicated bacterial growth (+), whereas clarity with unchanged OD indicated no growth (–). The lowest concentration of WEO that maintained clarity was defined as the MIC.

To determine the minimum bactericidal concentration (MBC), 100 μL from all non-turbid MIC tubes was subcultured onto fresh LB agar plates. After incubation at 37 °C for 24 h, the MBC was the showed no colony growth (Meng et al., 2022).

### 2.10 Bacterial growth inhibition curves

Bacterial suspensions of *E. coli* and *S. aureus* were adjusted to 1 × 10^6^ CFU/mL with sterile saline. Aliquots were inoculated into tubes containing LB broth supplemented with WEO at subinhibitory (1/2 MIC) and MIC concentrations. A control sample was prepared with an equivalent volume of sterile saline. All tubes were incubated at 37 °C with shaking at 100 rpm. At 0, 1, 2, 4, 6, 8, 10, 12, and 24 h, 100 μL samples were withdrawn, and the OD_600_ was measured using a microplate reader. Growth curves were plotted with incubation time on the x-axis and OD_600_ on the y-axis (Guo et al., 2024).

### 2.11 Investigation of antibacterial mechanisms

#### 2.11.1 Effect on conductivity of bacterial suspension

The effect of WEO on bacterial membrane permeability was assessed by measuring suspension conductivity (25 ± 1 °C). *E. coli* and *S. aureus* were cultured to mid-log phase, harvested by centrifugation (10,000 r/min for 10 min), and washed three times with sterile PBS. The cell pellets were resuspended in PBS to 1 × 10^6^ CFU/mL. Aliquots (15 mL) were treated with WEO at MIC and 2 MIC. A control received without was WEO was included. All samples were incubated at 37 °C with shaking (100 rpm) for up to 10 h. At 2 h intervals, 3 mL was sampled, and the electrical conductivity was measured. Conductivity was monitored throughout the incubation, with each treatment analyzed in triplicate (Yuan et al., 2024). Relative conductivity was calculated using Equation (2):


$$\mathrm{Relative}\mathrm{conductivity}\left(\%\right)=\frac{{\text{C}}_{\text{t}}-{\text{C}}_{0}}{C-{\text{C}}_{0}}\times 100\%$$ (2)

where C_0_ (mS/cm) is the conductivity of the sterile PBS at 0 h, C_*i*_ (mS/cm) is the conductivity of the bacterial suspension at time t, and C (mS/cm) is the conductivity of the bacterial suspension after complete cell lysis by boiling.

#### 2.11.2 Determination of nucleic acid and protein leakage

Bacterial suspensions were prepared as described in section 2.11.1. Aliquots (10 mL) were treated with WEO at MIC and 2 MIC; a sample without WEO served as the control. After incubation at 37 °C with shaking (100 rpm) for 10 h, 2 mL samples were collected every 2 h and centrifuged at 4 °C (1,000 rpm, 5 min). The supernatants were transferred to a microplate, and the OD_260_ and OD_280_ were recorded. Temporal changes in OD_260_ and OD_280_ were used to quantify the leakage of intracellular nucleic acids and proteins, respectively (Yi et al., 2024). All experiments were performed in triplicate.

#### 2.11.3 Assessment of cell membrane integrity by propidium iodide staining

*E. coli* and *S. aureus* cultures in mid-log phase were harvested by centrifugation (8,000 rpm, 10 min). The pellets were resuspended and treated with WEO at 0 (control), MIC, and 2 MIC for 24 h at 37 °C with shaking (100 rpm). After treatment, cells were collected by centrifugation (8,000 rpm, 5 min), washed once with PBS, and then incubated with propidium iodide (PI, 10 μg/mL) in the dark at room temperature for 20 min. The stained cells were washed three times with PBS, resuspended in a small volume of PBS, and examined under a fluorescence microscope. PI-positive cells (red fluorescence) indicated compromised membrane integrity (Li C. Z. et al., 2022).

#### 2.11.4 Assessment of cell wall integrity via alkaline phosphatase activity

Extracellular alkaline phosphatase (AKP) activity was measured as an indicator of bacterial cell wall damage. Bacterial suspensions (1 × 10^6^ CFU/mL) were treated with WEO at MIC and 2 MIC and incubated at 37 °C with shaking (180 rpm) for 8 h; a control received sterile water. After incubation, suspensions were centrifuged (1,000 rpm, 5 min, 4 °C). Supernatant AKP activity was quantified using a commercial assay kit according to the manufacturer’s instructions (Li J. et al., 2022).

#### 2.11.5 Scanning electron microscopy (SEM)

*E. coli* and *S. aureus* cells in mid-log phase were adjusted to 1 × 10^6^ CFU/mL and treated with WEO at 2 MIC for 8 h at 37 °C. Cells were harvested by centrifugation (8,000 rpm, 10 min), washed three times with PBS (0.1 M, pH 7.4), and fixed with 2.5% glutaraldehyde at 4 °C for 4 h. After fixation, samples were dehydrated through a graded ethanol series (30, 50, 70, 90, and 100%). The dehydrated samples were freeze-dried and sputter-coated with gold. Morphological alterations were observed using a scanning electron microscope (Samiah et al., 2023).

### 2.12 Statistical analysis

All experiments were performed in triplicate. Data are expressed as mean ± SD. One-way ANOVA followed by Duncan’s test was used to determine significant differences among groups (*p* < 0.05). Response surface design and model analysis were conducted using Design-Expert 13 software. Volatile oil components were identified by comparing mass spectra with the NIST library (match factor ≥ 80), with further confirmation based on retention times and Kovats retention indices. Relative quantification was performed using peak area normalization. Graphs were generated using Origin, and statistical calculations were performed with SPSS.

## 3 Results

### 3.1 Results of single-factor experiments

#### 3.1.1 Effect of electric field intensity on WEO yield

**FIGURE 1:**
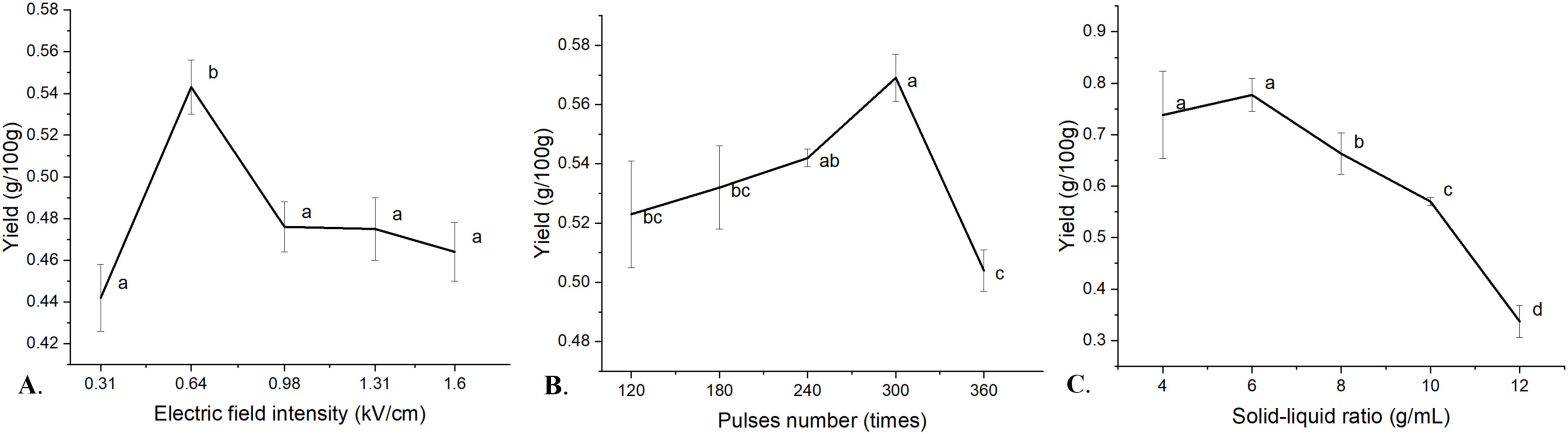
Effects of electric field intensity, pulse number and solid-liquid ratio on the yield of volatile oil from *W. villosa* leaves. **(A)** Electric field intensity. **(B)** Pulses number. **(C)** Solid-liquid ratio. Different lowercase letters indicate significant differences in the yield of volatile oil from *W. villosa* leaves under different levels (*P* < 0.05), while the same letter indicates no significant difference (*P* > 0.05).

The volatile oil yield increased with electric field intensity up to 0.64 kV/cm, peaking at 0.543 g/100 g (Figure 1A). Beyond this optimum, the yield declined significantly, representing a decrease of approximately 12.34% at 1.6 kV/cm compared to the peak value. This trend is explained by electroporation: a higher field enhances the transmembrane potential, inducing pore formation upon exceeding a critical threshold and thereby releasing intracellular volatile oils (Zhou et al., 2021). However, near or above 0.64 kV/cm, membrane permeabilization likely saturates. Further increases generate excessive Joule heating (Zhao et al., 2022), potentially degrading thermolabile compounds and reducing yield. Thus, 0.64 kV/cm was identified as the optimal electric field intensity.

#### 3.1.2 Effect of pulse number on WEO yield

Volatile oil yield as a function of pulse number is shown in Figure 1B. The yield increased up to 300 pulses, reaching a maximum of 0.569 g/100 g. Beyond 300 pulses, the yield decreased by approximately 11.42% at 360 pulses. This biphasic response may be due to competing mechanisms. An optimal number of pulses is expected to enhance cumulative electroporation, thereby improving membrane permeability and mass transfer (Xu et al., 2024; Ye et al., 2024). Conversely, excessive pulses cause significant thermal accumulation and electrochemical side reactions, degrading volatile constituents and reducing yield (Zhao et al., 2022). Therefore, 300 pulses was determined to be optimal.

#### 3.1.3 Effect of solid-liquid ratio on WEO yield

The volatile oil yield showed a parabolic dependence on the solid-liquid ratio (Figure 1C). A maximum yield of 0.777 g/100 g was obtained at a ratio of 1:6 g/mL. When the ratio was increased to 1:12 g/mL, the yield decreased sharply to 0.337 g/100 g. This represents a reduction of approximately 56.6% from the maximum yield. This trend is attributed to the interplay between mass transfer and energy distribution. At the optimum ratio (1:6 g/mL), the higher solid concentration favors mass transfer via an enhanced osmotic gradient and increases electrical conductivity, synergistically improving electroporation efficiency (Ye et al., 2024). Conversely, an excessive liquid volume dilutes the electric field energy per unit mass, reducing the effective treatment intensity and thus the extraction efficiency (Zhao et al., 2022). Hence, a solid-liquid ratio of 1:6 g/mL was optimal.

### 3.2 Optimization of PEF-HD extraction by box-Behnken design

Based on single-factor experiments, Electric field intensity (A), Pulse number (B), and Solid-liquid ratio (C) were selected as independent variables, with volatile oil yield as the response. A three-factor, three-level Box–Behnken design (BBD) was employed for process optimization. The experimental design matrix and corresponding responses are listed in Table 2.

**TABLE 2:** Experimental design matrix and responses of the Box-Behnken design.

Number	A: Electric field intensity (kV/cm)	B: Pulse number (times)	C: Solid-liquid ratio (g/mL)	Yield (g/100 g)
1	0.31	240	1:6	0.636
2	0.98	240	1:6	0.610
3	0.31	360	1:6	0.585
4	0.98	360	1:6	0.551
5	0.31	300	1:4	0.490
6	0.98	300	1:4	0.541
7	0.31	300	1:8	0.525
8	0.98	300	1:8	0.507
9	0.645	240	1:4	0.557
10	0.645	360	1:4	0.549
11	0.645	240	1:8	0.634
12	0.645	360	1:8	0.539
13	0.645	300	1:6	0.757
14	0.645	300	1:6	0.745
15	0.645	300	1:6	0.767

The data from Table 2 were fitted to a second-order polynomial model using Design-Expert 13 software. The model describes the relationship between the WEO yield (Y, g/100 g) and the independent variables: Electric field intensity (A, kV/cm), Pulse number (B, times), and Solid-liquid ratio (C, g/mL): Y = 0.7563 – 0.0034 A – 0.00266 B + 0.0085 C – 0.0020 AB – 0.0173 AC – 0.0218 BC – 0.1074 A^2^ – 0.0534 B^2^ – 0.1332^2^

The analysis of variance (ANOVA) results for the quadratic model are shown in Table 3. The model was highly significant (*F* = 34.05, *p* < 0.01). The lack-of-fit was not significant (*p* = 0.1878), confirming the model’s adequacy for fitting the data and for prediction. The model showed a high goodness-of-fit, with *R*^2^ = 0.9839 and adjusted *R*^2^ = 0.9551, indicating it effectively explains the response variation. Coefficient significance testing revealed that the quadratic terms (A^2^, B^2^, C^2^) had highly significant effects, while the linear term of pulse number (B) and the AC interaction term were also significant. The relative influence of the variables followed the order: B > C > A. In summary, the developed model is statistically robust and suitable for optimizing the PEF-HD process for WEO extraction.

**TABLE 3:** Response surface regression model and variance analysis results.

Source	Sum of squares	df	Mean square	*F*-value	*P*-value	
Model	0.1149	9	0.0128	34.05	0.0006**	Significant
A- Electric field intensity	0.0001	1	0.0001	0.2431	0.6429	
B- Pulse number	0.0057	1	0.0057	15.13	0.0115*	
C- Solid-liquid ratio	0.0006	1	0.0006	1.54	0.2694	
AB	0.0001	1	0.0001	0.0427	0.8445	
AC	0.0012	1	0.0012	3.17	0.1349	
BC	0.0019	1	0.0019	5.05	0.0746	
A^2^	0.0426	1	0.0426	113.64	0.0001**	
B^2^	0.0105	1	0.0105	28.10	0.0032**	
C^2^	0.0655	1	0.0655	174.66	<0.0001**	
Residual	0.0019	5	0.0004			
Lack of fit	0.0016	3	0.0005	4.48	0.1878	Not significant
Pure error	0.0002	2	0.0001			
Cor total	0.1168	14				
*R* ^2^	0.9839			R^2^_*Adj*_	0.9551	

** Denotes extremely significant influence, *P* < 0.01; * denotes significant influence, *P* < 0.05.

**FIGURE 2:**
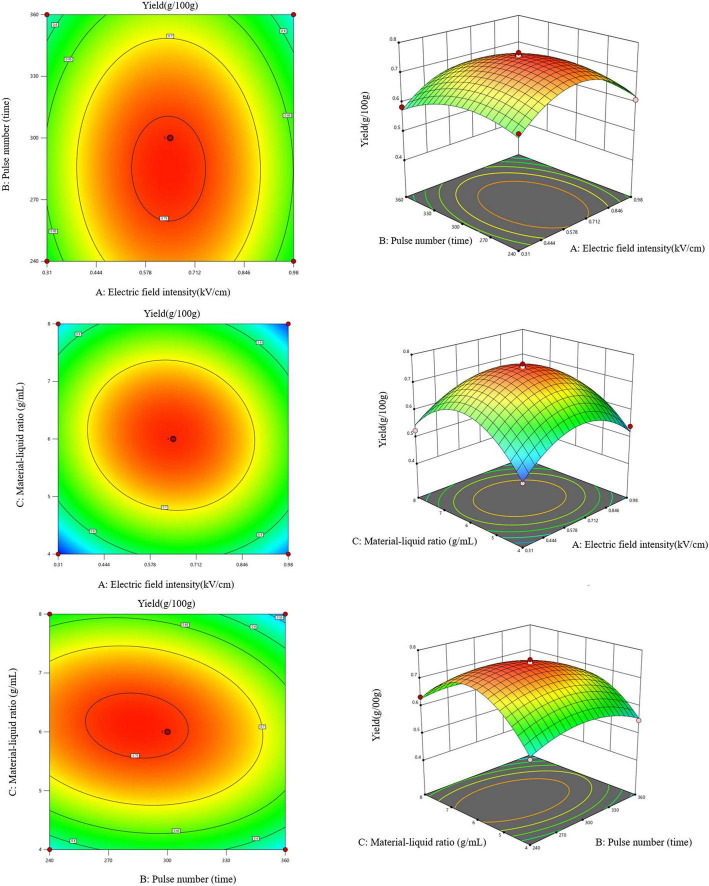
Response surface and contour plots illustrating the interactive effects of the pulsed electric field parameters: **(A)** electric field intensity and pulse number; **(B)** electric field intensity and solid-liquid ratio; **(C)** pulse number and solid-liquid ratio.

The pairwise interactions among electric field intensity (A), pulse number (B), and solid-liquid ratio (C) are visualized in the contour and response surface plots in Figure 2. The interaction between A and B was the weakest (Figure 2A). A moderate interaction was evident between A and C (Figure 2B). In contrast, the B–C interaction was the most pronounced (Figure 2C). Specifically, the yield showed a parabolic trend with increasing B and C, consistent with the earlier single-factor results.

#### 3.2.1 Validation of optimized PEF-HD conditions

Based on the model analysis, the optimal conditions were adjusted for practical application: electric field intensity of 0.64 kV/cm, pulse number of 284, and solid-liquid ratio of 1:6 g/mL. Three validation experiments under these optimal conditions yielded an average WEO yield of 0.779 ± 0.034 g/100 g. This yield exceeded the best single-factor result (0.777 g/100 g), closely matched the model prediction (0.760 g/100 g; deviation: 0.025 g/100 g), and represented a 1.32-fold increase over the traditional method (0.588 ± 0.089 g/100 g). For comparison, Barros et al. reported a 40% higher extraction rate using a 1 kV/cm field with 0.4 kJ/kg and 30 min distillation (Barros et al., 2022). Similarly, Miloudi et al. showed that PEF achieved in 60 min the efficiency requiring 120 min traditionally (Kaddour et al., 2022). These validation results indicate that the RSM model is accurate and reliable for predicting the optimal PEF-HD conditions for WEO extraction.

### 3.3 Comparative analysis of the microstructure of *W. villosa* leaves

**FIGURE 3:**
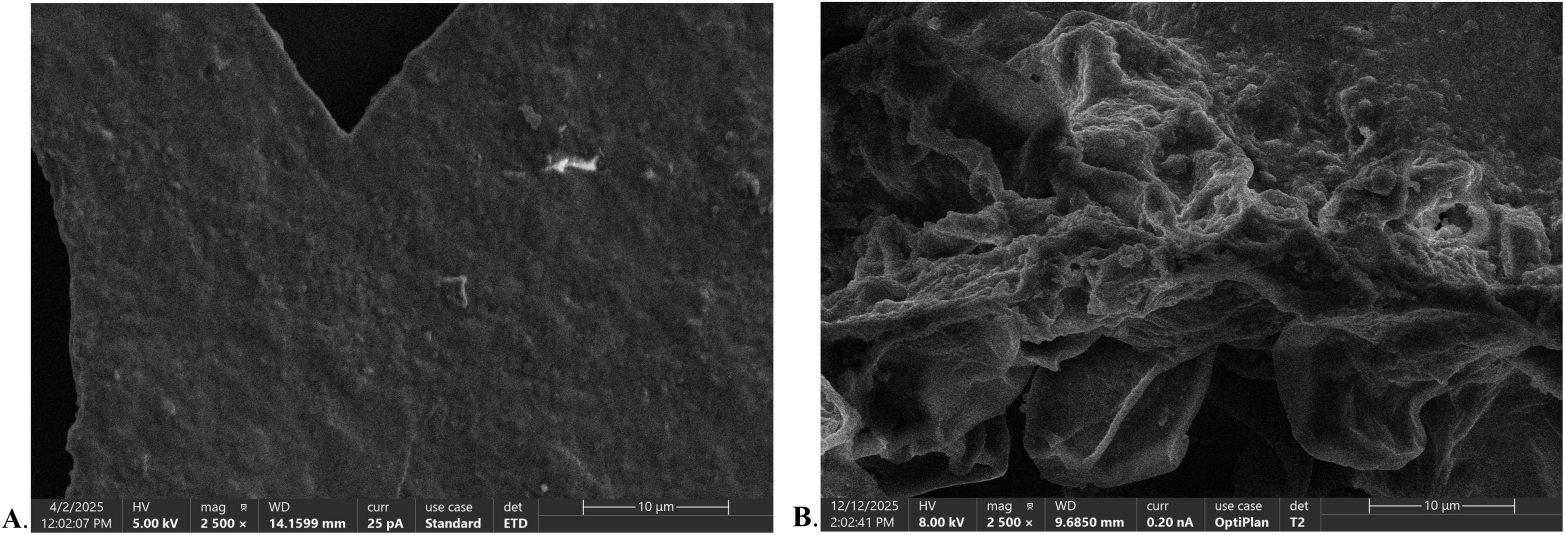
Microstructure of the surface of *W. villosa* leaves. **(A)** Untreated leaf surface. **(B)** Leaf surface after PEF pretreatment.

The micro-morphology of *W. villosa* leaves before and after PEF treatment was examined by scanning electron microscopy (SEM), and representative images are shown in Figure 3. The surface of untreated leaves appeared structurally intact and smooth (Figure 3A). After PEF treatment, the leaf surface exhibited significant structural alterations, including cell membrane damage and extensive shrinkage (Figure 3B). The physical action of PEF induced uneven contraction, resulting in a rough, multi-folded surface texture. Distinct undulating, gully-like patterns emerged, and fine particles were scattered across the surface.

### 3.4 Chemical composition analysis of WEO

The chemical composition of WEO from *W. villosa* leaves extracted by PEF-HD was analyzed using GC–MS. In total, 59 compounds were identified and categorized into five major classes (Table 4): terpenes (16 compounds, 66.74%), oxygenated terpenoids (27 compounds, 25.82%), aliphatic compounds (8 compounds, 3.47%), aromatic/benzene-ring derivatives (4 compounds, 3.11%), and others (4 compounds, 0.86%). Terpenes and oxygenated terpenoids collectively accounted for 92.56% of the total oil, representing the principal constituents.

**TABLE 4:** Chemical composition of volatile oil from *W. villosa* leaves (WEO) extracted by PEF-HD.

No.	RT	Name	Structural formula	CAS	KI	KI*	Relative amount (%)
Terpene							
1	19.24	Camphene	C_10_H_16_	79-92-5	1005	1043	1.459 ± 0.542
2	22.12	β-Phellandrene	C_10_H_16_	555-10-2	1070	1183	29.951 ± 5.371
3	23.76	(1S)-(1)-β-Pinene	C_10_H_16_	18172-67-3	1125	1118	7.138 ± 1.016
4	24.71	Terpilene	C_10_H_16_	99-86-5	1172	1178	4.540 ± 0.301
5	25.73	D-Limonene	C_10_H_16_	5989-27-5	1206	/	9.022 ± 0.911
6	27.24	trans-β-Ocimene	C_10_H_16_	3779-61-1	1228	1247	0.770 ± 0.190
7	27.95	γ-Terpinene	C_10_H_16_	99-85-4	1237	1243	8.536 ± 1.001
8	29.84	(+)-4-Carene	C_10_H_16_	29050-33-7	1264	1149	2.636 ± 0.308
9	34.01	neo-Alloocimene,stab.	C_10_H_16_	7216-56-0	1325	1370	0.552 ± 0.085
10	37.38	p-Mentha-1,5,8-triene	C_10_H_14_	21195-59-5	1376	1375	0.101 ± 0.013
11	37.79	1,3,8-p-Menthatriene	C_10_H_14_	18368-95-1	1383	/	0.096 ± 0.014
12	39.65	γ-Elemene	C_15_H_24_	29873-99-2	1425	1434	0.288 ± 0.021
13	44.25	Bicyclo[5.2.0]nonane, 2-methylene-4,8,8-trimethyl-4-vinyl-	C_15_H_24_	242794-76-9	1561	/	0.951 ± 0.089
14	47.31	Aristolochene	C_15_H_24_	26620-71-3	1707	1669	0.279 ± 0.020
15	48.21	(+)-Bicyclogermacrene	C_15_H_24_	24703-35-3	1756	1752	0.195 ± 0.024
16	48.53	(+)-epi-Bicyclosesquiphellandrene	C_15_H_24_	54274-73-6	1775	1760	0.181 ± 0.029
Oxygen-containing terpenoids							
17	31.95	Cyclohexanone, 2,2,6-trimethyl-	C_9_H_16_O	2408-37-9	1293	1282	0.048 ± 0.009
18	36.32	3,6,6-Trimethyl-2-Cyclohexen-1-One	C_9_H_14_O	23438-77-9	1360	1366	0.028 ± 0.003
19	38.18	Thujone	C_10_H_16_O	471-15-8	1389	/	0.070 ± 0.011
20	41.43	Ethanone, 1-(1,4-dimethyl-3-cyclohexen-1-yl)-	C_10_H_16_O	43219-68-7	1485	1491	0.325 ± 0.035
21	41.57	Linalool	C_10_H_18_O	78-70-6	1490	1502	2.575 ± 1.164
22	42.08	( ± )-trans-4-Thujanol	C_10_H_18_O	17699-16-0	1505	1483	0.118 ± 0.012
23	42.29	trans-2-Pinanol	C_10_H_18_O	4948-29-2	1511	1432	0.046 ± 0.006
24	42.66	Isopinocamphone	C_10_H_16_O	15358-88-0	1520	1555	1.215 ± 0.073
25	43.28	Fenchol	C_10_H_18_O	1632-73-1	1536	1543	0.909 ± 0.072
26	43.37	Pinocarvone	C_10_H_14_O	30460-92-5	1549	1566	1.244 ± 0.071
27	43.82	(-)-Isopinocampheol, acetate	C_12_H_20_O_2_	1000462-98-1	1549	/	0.777 ± 0.109
28	44.02	terpinen-4-ol	C_10_H_18_O	562-74-3	1555	1552	6.291 ± 0.418
29	44.65	2-Cyclohexen-1-ol, 1-methyl-4-(1-methylethyl)-, cis-	C_10_H_18_O	29803-82-5	1570	1563	0.195 ± 0.010
30	44.86	β-Cyclocitral	C_1_0H_16_O	432-25-7	1576	1586	0.083 ± 0.008
31	45.01	α-Thujenal	C_10_H_14_O	57129-54-1	1579	/	0.069 ± 0.004
32	45.23	Myrtenal	C_10_H_14_O	564-94-3	1585	1597	2.199 ± 0.073
33	45.43	Bicyclo[3.1.0]hex-3-en-2-one, 4-methyl-1-(1-methylethyl)-	C_10_H_14_O	24545-81-1	1590	1614	0.102 ± 0.005
34	45.59	Bicyclo[3.1.1]hept-3-en-2-ol, 4,6,6- trimethyl-, [1S-(1α,2β,5α)]-	C_10_H_16_O	18881-04-4	1593	1645	0.054 ± 0.002
35	46.23	trans-Verbenol	C_10_H_16_O	1820-09-3	1630	1648	0.273 ± 0.024
36	46.63	L-α-Terpineol	C_10_H_18_O	10482-56-1	1659	1690	4.391 ± 0.146
37	46.94	endo-Borneol	C_10_H_18_O	507-70-0	1682	1698	1.302 ± 0.068
38	47.35	p-Mentha-1,5-dien-8-ol	C_10_H_16_O	1686-20-0	1709	1714	0.378 ± 0.018
39	47.55	cis-p-mentha-1(7),8-dien-2-ol	C_10_H_16_O	1000374-16-8	1720	1774	0.181 ± 0.099
40	47.65	Neral	C_10_H_16_O	106-26-3	1726	1733	0.254 ± 0.035
41	49.56	(-)-Myrtenol	C_10_H_16_O	19894-97-4	1815	1807	2.549 ± 0.148
42	50.64	2-Caren-4-ol	C_10_H_16_O	6617-35-2	1843	1816	0.064 ± 0.009
43	51.23	.alpha.-Ionone	C_13_H_20_O	127-41-3	1859	1863	0.088 ± 0.016
Aliphatic compounds							
44	23.51	1-Penten-3-ol	C_5_H_10_O	616-25-1	1110	1112	0.311 ± 0.076
45	32.26	5-Hepten-2-one, 6-methyl-	C_8_H_14_O	110-93-0	1297	1317	0.077 ± 0.016
46	32.88	1-Hexanol	C_6_H_14_O	111-27-3	1307	1325	0.109 ± 0.028
47	34.51	3-Hexen-1-ol	C_6_H_12_O	928-96-1	1332	1351	0.246 ± 0.067
48	35.13	Nonanal	C_9_H_18_O	124-19-6	1342	1348	0.036 ± 0.005
49	37.24	1-Undecene, 9-methyl-	C_12_H_24_	74630-41-4	1374	1392	0.031 ± 0.004
50	46.75	( ± )-methyl myrtenate	C_11_H_16_O_2_	30649-97-9	1668	1670	2.261 ± 0.143
51	49.43	Aspirin methyl ester	C_10_H_10_O_4_	580-02-9	1812	1822	0.396 ± 0.023
Aromatic/benzene ring derivatives							
52	29.18	o-Cymene	C_10_H_14_	527-84-4	1254	1276	2.120 ± 0.329
53	37.62	Benzene, (2-methyl-1-propenyl)-	C_10_H_12_	768-49-0	1380	/	0.224 ± 0.027
54	41.49	Benzaldehyde	C_7_H_6_O	100-52-7	1487	1480	0.387 ± 0.276
55	49.68	Benzene, 1-methyl-3-(1-methylethyl)-	C_10_H_14_	535-77-3	1818	1264	0.428 ± 0.049
Other							
56	40.49	Dihydroedulan II	C_13_H_22_O	41678-32-4	1453	1492	0.179 ± 0.030
57	40.84	1-Oxaspiro[4.5]dec-6-ene, 2,6,10,10-tetramethyl-	C_13_H_22_O	36431-72-8	1465	1482	0.143 ± 0.025
58	41.95	Cyclohexane, 2-ethenyl-1,1-dimethyl-3-methylene-	C_11_H_18_	95452-08-7	1502	/	0.216 ± 0.019
59	50.77	2-(4-Methylphenyl)propan-2-ol	C_10_H_14_O	1197-01-9	1847	1852	0.311 ± 0.030

RT, Retention time; KI, Calculated retention index; KI*, Polar column retention index (retrieved from PubMed database).

As the predominant components, terpenes consist mainly of monoterpenes (C_10_). The five most abundant monoterpenes were β-phellandrene (29.95%), D-limonene (9.02%), γ-terpinene (8.46%), β-Pinene (7.14%), and 4-carene (2.64%). These compounds are highly hydrophobic and volatile, contributing significantly to the characteristic aroma and bioactivities of the oil. Oxygenated terpenoids, the second major class, primarily comprise terpene alcohols, ketones, and aldehydes. The most abundant alcohols were 4-terpineol (6.29%) and α-terpineol (4.39%), followed by linalool (2.58%) and myrtenol (2.55%). The major ketones were Pinocarvone (1.24%) and isopinocamphone (1.22%), while myrtenal (2.20%) was the predominant aldehyde. The oxygen-containing functional groups generally increase polarity and enhance bioactivity. Notable non-terpenoid compounds include methyl myristate (2.26%) among aliphatics and o-cymene (2.12%) among aromatics.

### 3.5 Antibacterial activity of WEO against *E. coli* and *S. aureus*

#### 3.5.1 Antibacterial activity assessed by disk diffusion

The inhibition zone diameter directly indicates antibacterial efficacy. Bacterial sensitivity was classified based on inhibition zone diameter (Shu et al., 2025): not sensitive (–, ≤ 8 mm), sensitive (+, 8–14 mm), and highly sensitive (++, 14–20 mm). WEO-impregnated discs produced clear inhibition zones against both *E. coli* (11.62 ± 0.45 mm) and *S. aureus* (13.30 ± 0.30 mm) in Table 5. The positive control (ampicillin) yielded zones of 25.27 ± 0.35 mm for *E. coli* and 20.77 ± 1.11 mm for S. aureus. The results demonstrated that WEO inhibited both tested bacterial strains.

**TABLE 5:** Bacteriostatic circle.

Culture	WEO	AMP
*E. coli*	11.62 ± 0.45 mm	25.27 ± 0.35 mm
*S. aureus*	13.30 ± 0.30 mm	20.77 ± 1.11 mm

#### 3.5.2 Determination of minimum inhibitory concentration (MIC) and minimum bactericidal concentration (MBC)

The minimum inhibitory concentration (MIC) and minimum bactericidal concentration (MBC) are key parameters for assessing antimicrobial potency. The MIC and MBC of WEO against *E. coli* and *S. aureus* were determined using the two-fold dilution method. The MIC values of WEO were 3.2 mg/mL for *E. coli* and 1.6 mg/mL for *S. aureus* (Table 6). After 24 h of incubation, the solutions in the MIC tubes remained clear, with OD_600_ values showing no difference from the blank control, whereas the negative control was turbid with an increase in OD_600_, indicating inhibition of bacterial growth. To evaluate bactericidal activity, suspensions treated with WEO for 24 h were subcultured onto agar plates. For *E. coli*, the solution remained clear with unchanged OD_600_ at WEO concentrations of ≥ 3.2 mg/mL, and no colonies were observed after subculturing at 6.4 mg/mL, establishing an MBC of 6.4 mg/mL. For *S. aureus*, the solution remained clear with no increase in OD_600_ at concentrations of ≥ 1.6 mg/mL, and no colonies grew after subculturing at 3.2 mg/mL, corresponding to an MBC of 3.2 mg/mL. Compared with other plant-derived volatile oils, *ginger* volatile oil exhibited MIC values of 2.0 mg/mL against *E. coli* and 1.0 mg/mL against *S. aureus*, and MBC values of 4.0 mg/mL and 2.0 mg/mL, respectively (Wang et al., 2020). The volatile oil from the aerial parts of *Levisticum officinale* W.D.J. Koch showed MIC values of 2.0 mg/mL against *S. aureus* and 4.0 mg/mL against *E. coli* (Miran et al., 2018). These results indicate that WEO exhibits bactericidal activity against both tested strains.

**TABLE 6:** Minimum inhibitory concentration (MIC) and minimum bactericidal concentration (MBC) of WEO against *E. coli* and *S. aureus.*

Strain	Concentration (mg/mL)	51.2	25.6	12.8	6.4	3.2	1.6
*E. coli*	MIC	−	−	−	−	+	+
	MBC	−	−	−	+	+	+
*S. aureus*	MIC	−	−	−	−	−	+
	MBC	−	−	−	−	+	+

“+” indicates visible turbidity (bacterial growth); “-” indicates clarity (no growth).

#### 3.5.3 Bacterial growth inhibition curves

**FIGURE 4:**
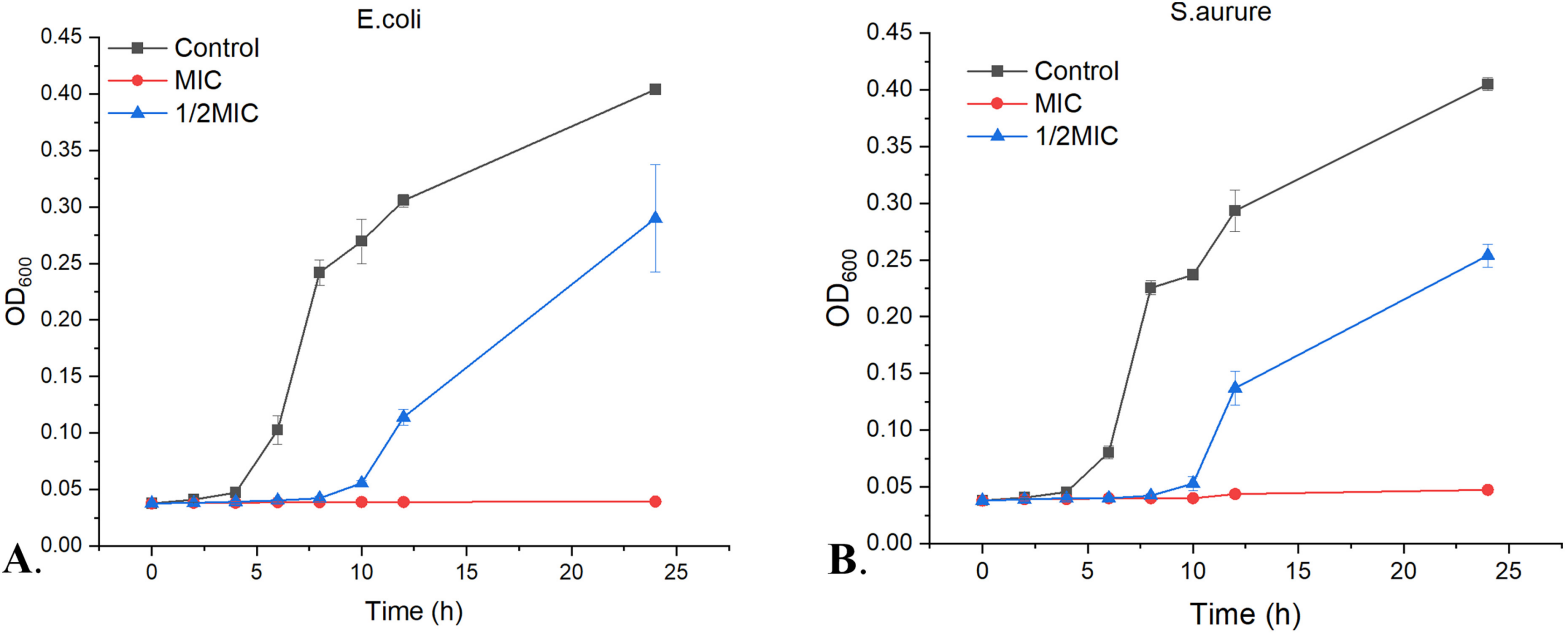
Inhibitory effects of WEO on the growth of *E. coli* and *S. aureus*. **(A)** Growth curve of *E. coli*. **(B)** Growth curve of *S. aureus*.

The growth kinetics of *E. coli* and *S. aureus* were evaluated at different WEO concentrations, with untreated cultures as negative controls. WEO significantly altered the growth profiles of both bacteria. At the MIC, WEO completely suppressed the growth of both strains over 24 h (Figures 4A,B). At a subinhibitory concentration (1/2 MIC), the OD_600_ of both bacteria remained stable during the first 0–8 h, indicating a bacteriostatic effect. However, upon prolonged incubation, growth resumed at a rate initially slower than the control before accelerating. The growth rates of both bacteria were significantly reduced by WEO in a concentration-dependent manner. At equivalent concentrations, growth inhibition was more pronounced against *S. aureus* than *E. coli*. This difference may stem from their distinct cell wall structures: *E. coli*, a Gram-negative bacterium, has an outer membrane, a thin peptidoglycan layer, and a periplasmic space, collectively forming a more effective barrier against hydrophobic agents. In contrast, *S. aureus*, a Gram-positive bacterium, has a thick peptidoglycan layer more susceptible to disruption by hydrophobic oil components (Dannenberg et al., 2019). These results demonstrate that WEO significantly inhibits and delays the proliferation of both *E. coli* and *S. aureus*.

### 3.6 Effects of WEO on the cellular integrity of *E. coli* and *S. aureus*

#### 3.6.1 Effect of WEO on the extracellular conductivity of *E. coli* and *S. aureus* suspensions

**FIGURE 5:**
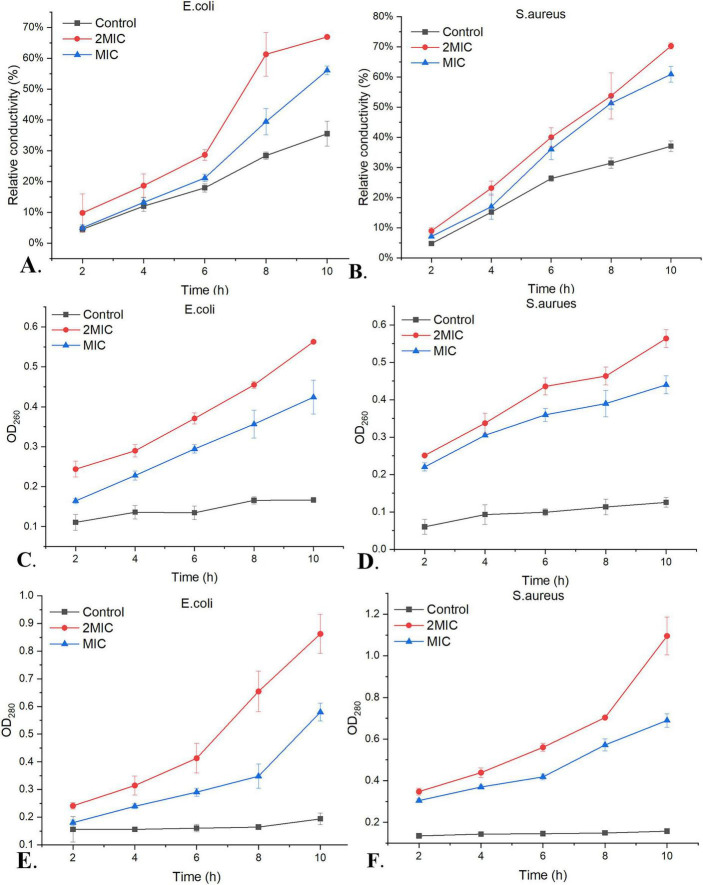
Effects of WEO on extracellular conductivity, nucleic acids, and proteins of *E. coli* and *S. aureus* membranes **(A)** Effect of WEO on the conductivity of *E. coli*. **(B)** Effect of WEO on the conductivity of *S. aureus*. **(C)** Effect of WEO on nucleic acids of *E. coli*. **(D)** Effect of WEO on nucleic acids of *S. aureus*. **(E)** Effect of WEO on proteins of *E. coli*. **(F)** Effect of WEO on proteins of *S. aureus*).

Relative conductivity reflects bacterial membrane permeability, with higher values indicating greater membrane damage. Treatment with MIC and 2 MIC WEO significantly altered relative conductivity within 10 h (Figure 5), with rapid increases compared to the untreated control. After 10 h, relative conductivity reached 66.96 ± 0.67% (*E. coli*) and 70.29 ± 1.02% (*S. aureus*) at 2 MIC, and 56.10 ± 1.39% and 60.91 ± 2.61% at MIC (Figures 5A,B). Compared with the control, this represented a 0.89-fold (*E. coli*) and 0.90-fold (*S. aureus*) increase at 2 MIC, and 0.58-fold and 0.64-fold at MIC. In this study, WEO treatment significantly increased the relative conductivity of *E. coli* and *S. aureus* suspensions. This finding aligns with previous reports showing that volatile oil from *Artemisia giraldii Pamp.* disrupts membrane permeability and causes electrolyte leakage in these bacteria (Huo et al., 2022), and that *clove* leaf and bud oils affect the relative conductivity of *S. aureus* and *P. aeruginosa* (Samiah et al., 2023). Collectively, these results demonstrate that WEO damages both strains, causing electrolyte leakage and increasing suspension conductivity.

#### 3.6.2 Effect of WEO on the leakage of nucleic acids and proteins from *E. coli* and *S. aureus*

The bacterial cell membrane serves as a protective barrier and regulates nutrient transport to maintain normal growth. Damage to its integrity impairs barrier function, inhibiting growth and metabolism. Increased membrane permeability leads to the leakage of intracellular proteins (Zhao et al., 2025). The leakage of nucleic acids and proteins can be quantified by measuring extracellular optical density at their characteristic absorption peaks (260 nm and 280 nm, respectively) (Su et al., 2023). Following 10 h of treatment with MIC and 2 MIC WEO, OD_260_ values reached 0.424 and 0.563 for *E. coli*, and 0.440 and 0.564 for *S. aureus*, respectively (Figures 5C,D). Similarly, OD_280_ values for *E. coli* increased to 0.862 (2 MIC) and 0.580 (MIC), and for *S. aureus* to 1.10 and 0.689, respectively (Figures 5E,F). After 10 h, 2 MIC WEO treatment increased protein leakage by 3.25-fold (*E. coli*) and 6.00-fold (*S. aureus*), and nucleic acid leakage by 2.39- and 3.48-fold, respectively, versus the control. Similarly, it has been reported that the volatile oil from *Pinus pumila* seed scales also induces nucleic acid and protein leakage in *E. coli* (Mi et al., 2025). Together, these findings demonstrate that WEO disrupts membrane integrity, causing nucleic acid and protein leakage and consequently inhibiting bacterial growth.

#### 3.6.3 Assessment of bacterial membrane integrity by propidium iodide (PI) staining

**FIGURE 6:**
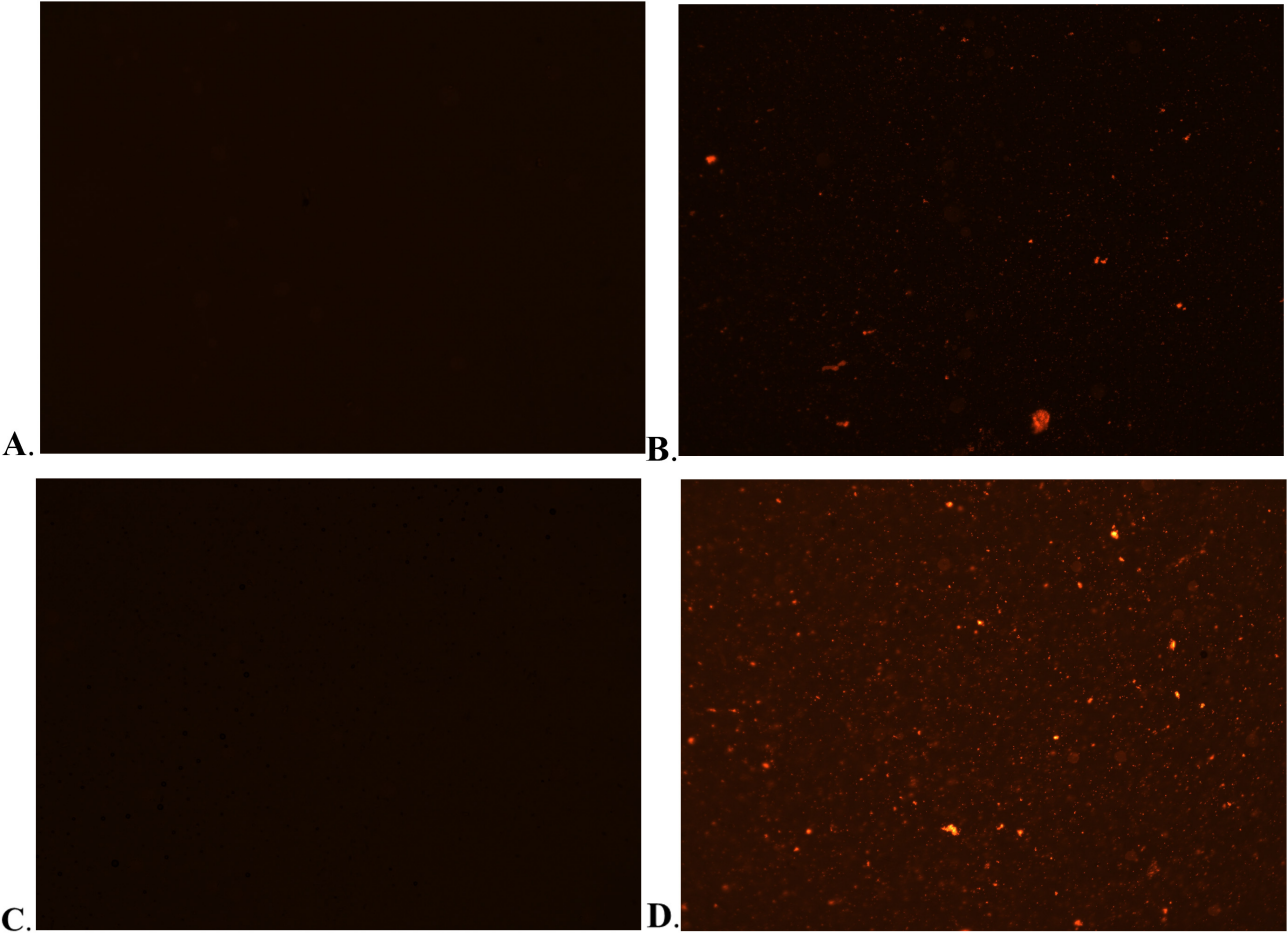
Effects of WEO on the cell membrane morphology of *E. coli* and *S. aureus*. **(A)** Untreated *E. coli*. **(B)** WEO-treated *E. coli*. **(C)** Untreated *S. aureus*. **(D)** WEO-treated *S. aureus*.

Propidium iodide (PI) is a membrane-impermeant fluorescent dye that selectively stains nucleic acids. It enters cells only upon membrane damage, binding to DNA and emitting red fluorescence (Huang et al., 2023). Consequently, PI staining is widely used to evaluate membrane integrity. PI staining was used to assess the effect of WEO on bacterial membrane integrity. Negligible red fluorescence was observed in untreated controls, confirming intact membranes that excluded the dye (Figures 6A,C). In contrast, 2 MIC WEO induced intense red fluorescence in both *E. coli* (Figure 6B) and *S. aureus* (Figure 6D), indicating compromised membrane integrity, enhanced permeability, and PI influx. Similarly, it has been reported that volatile oil from *Litsea cubeba* can penetrate the cell membrane of *E. coli* and cause structural damage (Yang et al., 2020). These findings further confirm that WEO impairs membrane integrity, facilitating dye penetration and underscoring its membrane-disruption mechanism.

#### 3.6.4 Effect on bacterial cell wall integrity via alkaline phosphatase (AKP) activity

**FIGURE 7:**
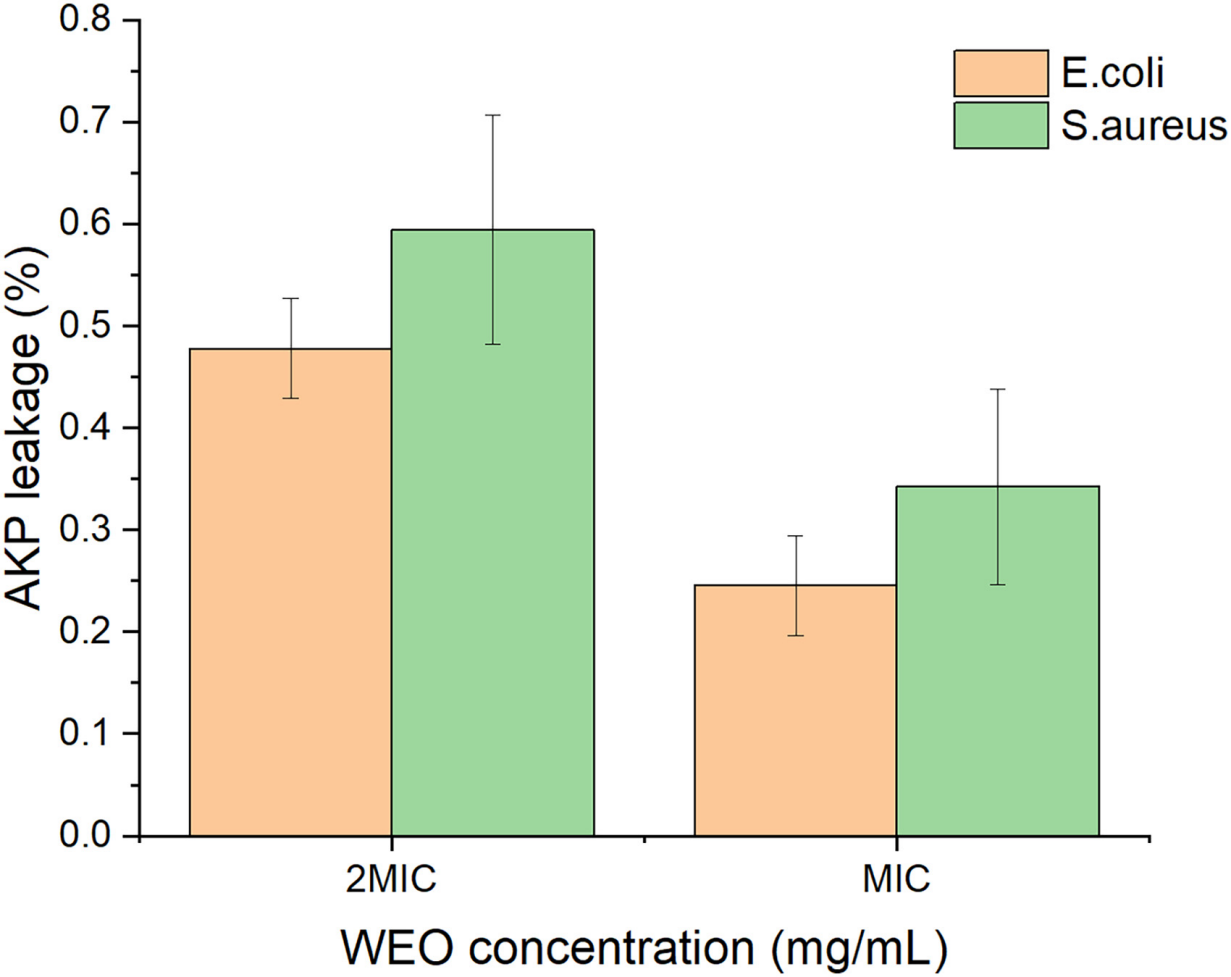
Effect of WEO on AKP activity in *E. coli* and *S. aureus*.

Alkaline phosphatase (AKP), an intracellular enzyme located between the cell wall and membrane, is a key indicator of cell wall integrity (Chen et al., 2025). Significant extracellular leakage of AKP occurs only upon cell wall rupture and loss of integrity (Souren et al., 2011). The extracellular AKP activity of *E. coli* and *S. aureus* treated with different WEO concentrations is shown in Figure 7. After treatment with MIC and 2 MIC WEO, the supernatant AKP activity was 24.56 and 47.83% for *E. coli*, and 34.26 and 59.49% for *S. aureus*, respectively. AKP activity increased with WEO concentration. These results indicate that WEO disrupts bacterial cell wall integrity, leading to AKP leakage and a consequent rise in extracellular activity.

#### 3.6.5 Scanning electron microscopy (SEM) observations

**FIGURE 8:**
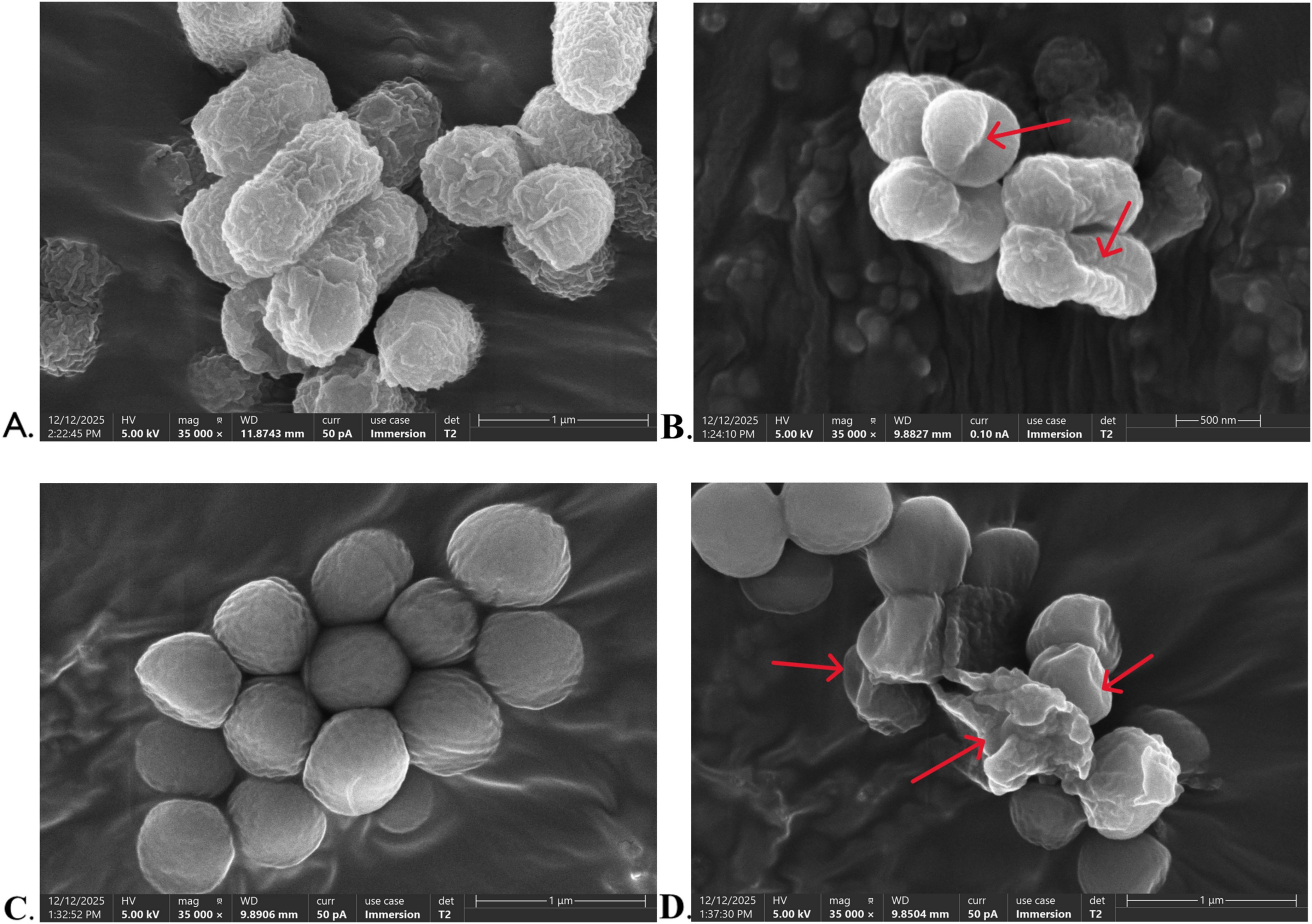
Scanning electron micrographs of *E. coli* and *S. aureus* treated with WEO. **(A)** *E. coli* without WEO treatment. **(B)** *E. coli* treated with WEO. **(C)** *S. aureus* without WEO treatment. **(D)** *S. aureus* treated with WEO.

Morphological changes in bacteria after treatment with 2 MIC WEO were examined by scanning electron microscopy (SEM) (Figure 8). Untreated *E. coli* exhibited a typical short rod-shaped morphology with intact cellular structure (Figure 8A), while untreated *S. aureus* displayed a regular spherical form with well-defined contours (Figure 8C). After WEO treatment, both species showed significant morphological alterations (Figures 8B,D), including surface shrinkage and depression, cell collapse or rupture, compromised cell wall and membrane integrity, and visible leakage of cytoplasmic content. These observations indicate that WEO targets the bacterial cell wall and membrane, leading to disruption, increased permeability, leakage of intracellular components, and ultimately cell death.

## 4 Discussion

This study utilized PEF-HD to extract WEO. PEF enhances extraction by inducing electroporation, disrupting plant cell structure and promoting the release of volatile oil components (Ferraz and Silva, 2025). Based on this principle, extraction parameters were optimized via single-factor experiments and response surface methodology. The chemical composition of the optimized WEO was analyzed by GC–MS, and its antibacterial activity against *E. coli* and *S. aureus* was evaluated. The results demonstrated antibacterial activity of WEO against both strains, with MBC values of 6.4 mg/mL for *E. coli* and 3.2 mg/mL for *S. aureus*. *S. aureus* was notably more susceptible to WEO than *E. coli*, likely due to the distinct permeability barriers: the thick peptidoglycan layer in Gram-positive bacteria versus the outer membrane in Gram-negative bacteria (Zhang et al., 2018).

The antibacterial mechanism of WEO stems from the chemical complexity of volatile oils, which hinders microbial resistance development, and their lipophilic nature, which facilitates active molecule diffusion to cellular targets (Hu et al., 2023). This study confirmed the close relationship between WEO’s antibacterial activity and its chemical composition. The abundant hydrophobic terpenes readily incorporate into and disrupt the bacterial lipid bilayer (Zengin and Baysal, 2014), significantly increasing permeability and causing leakage of proteins, nucleic acids, and electrolytes. Furthermore, oxygenated terpenoids with polar functional groups (e.g., hydroxyl) may interact with peptidoglycan or membrane proteins, exacerbating membrane damage and interfering with cell wall synthesis, as supported by the observed AKP leakage. PI staining and SEM imaging provided visual evidence that WEO induced substantial structural damage to bacterial membranes, leading to cellular collapse.

Among the main components of WEO, D-limonene and its isomers exert antimicrobial effects by disrupting the cell membrane, increasing membrane permeability, and interfering with respiratory metabolism (Akshi et al., 2021). They also inhibit spoilage fungi such as Candida tropicalis, leading to reduced membrane potential and compromised membrane integrity (Yu et al., 2022). Furthermore, D-limonene and β-phellandrene can inhibit the replication and expression of genetic material (Fan et al., 2025). Although studies on the antimicrobial activity of β-phellandrene alone are limited, essential oils rich in this compound have been shown to inhibit the growth of *Escherichia coli*, *Staphylococcus aureus*, and *Bacillus subtilis* (Amor et al., 2019; Zhang et al., 2022). γ-Terpinene inhibits the growth of Candida albicans and promotes its apoptosis (Asgar et al., 2025), while β-pinene is a natural compound with antifungal activity (Feng et al., 2020; Zhang et al., 2025). Notably, D-limonene and γ-terpinene can convert into oxygenated derivatives during storage, further enhancing their antimicrobial activity. Among oxygenated terpenes, α-terpineol directly damages the ultrastructure of the cell membrane, disrupts physiological functions, and induces DNA damage, thereby effectively inhibiting pathogens such as S. aureus and E. coli (Badawy et al., 2019; Zhu et al., 2025). 4-Terpinenol, in addition to compromising membrane integrity, inhibits bacterial biofilm formation (Laísa et al., 2020; Kerekes et al., 2019) and interferes with energy metabolism (Liu et al., 2025).

Moreover, the overall antibacterial efficacy of WEO likely arises from synergistic interactions among constituents such as α-terpineol, linalool, D-limonene and β-phellandrene. For example, α-terpineol and linalool have been reported to act synergistically by altering membrane permeability (Zengin and Baysal, 2014), while linalool disrupts pathogen membranes and interferes with basic metabolism (He M. W. et al., 2025; Li et al., 2025). Components like D-limonene and β-phellandrene may also act through multiple pathways, including affecting gene expression (Fan et al., 2025). In summary, the antibacterial action of WEO results from synergistic interactions: the physical disruption of membrane lipids by hydrophobic terpenes (e.g., β-phellandrene, D-limonene) and the chemical interactions of oxygenated terpenoids (e.g., α-terpineol, 4-terpineol, linalool) with membrane proteins and cell wall targets (Zengin and Baysal, 2014; Bianca et al., 2022; Santi et al., 2025). This multi-component, multi-target mode of action is analogous to other plant volatile oils, such as *oregano* volatile oil (Xia et al., 2025) and *Litsea cubeba* volatile oil (Hu et al., 2019), which primarily target membrane systems, and partly explains their lower propensity to induce specific bacterial resistance (Hu et al., 2023).

The potent inhibitory activity of WEO against *S. aureus* and *E. coli* provides a strong rationale for its application as a natural antimicrobial agent in functional foods. Foodborne pathogen contamination is a critical factor affecting food safety and human gut health. Representing Gram-positive and Gram-negative bacteria, respectively, *S. aureus* and *E. coli* are among the most common pathogens responsible for food spoilage and intestinal infections. The significant bactericidal activity of WEO against both strains highlights its potential to be developed as a food-derived natural antibacterial agent. Furthermore, WEO exerts its antibacterial effects through multi-target mechanisms, such as disrupting cell membrane integrity and inducing the leakage of intracellular contents. Compared to single-component antibiotics, this multi-target mode of action may help reduce the risk of bacterial resistance development (McCarthy and O’Gara, 2015). These properties highlight the potential of WEO as a potent, safe natural antimicrobial agent with a low risk of inducing bacterial resistance.

Although this study revealed the favorable *in vitro* antibacterial activity of WEO and its potential mechanisms, several limitations should be noted. First, the study was limited to two common bacterial strains. Future work should expand to include a broader range, such as clinically resistant bacteria and fungi, to fully assess its antimicrobial spectrum. Second, mechanistic investigations relied on phenotypic observations and literature analogies, lacking direct molecular evidence for key targets, such as specific metabolic pathways.

Despite these limitations, the results demonstrate that WEO exhibits effective antibacterial activity against *S. aureus* and *E. coli*. This activity may be attributed to the synergistic action of its terpenoid components, likely exerting a combined effect through multiple mechanisms, including membrane disruption, metabolic interference, and multi-component interactions.

## 5 Conclusion

This study employed PEF-HD to extract WEO. The extraction process was optimized using single-factor experiments and response surface methodology. The optimal parameters were: electric field strength of 0.64 kV/cm, pulse number of 284, and solid-liquid ratio of 1:6 g/mL. Under these optimal conditions, the WEO yield reached 0.779 ± 0.034 g/100 g, which represents a 1.32-fold increase over conventional hydrodistillation. GC–MS analysis identified 59 compounds, with terpenes as the predominant fraction (66.74%), followed by oxygenated terpenoids (25.82%). Mechanistic studies revealed that WEO exerts bactericidal effects through multi-target pathways, including disruption of bacterial cell membrane integrity and induction of intracellular content leakage. These findings demonstrate that PEF-HD is an efficient green technology for WEO extraction, yielding WEO rich in terpenoid components with antibacterial activity. This study provides a scientific basis and technical support for the development of *W. villosa* leaf resources as natural antibacterial agents.

This study provides preliminary validation of WEO’s inhibitory effect against representative bacterial strains, warranting further investigations to expand the evaluation of its antibacterial activity against a broader spectrum of food spoilage microorganisms and pathogens. In this context, WEO demonstrates promising potential as a food-derived natural antibacterial agent, owing to its bactericidal activity against *S. aureus* and *E. coli* and its multi-target antibacterial mechanism. This study provides a scientific basis for the high-value utilization of *W. villosa* leaf resources and the development of novel food-derived natural antibacterial agents. Future research should further investigate the stability of WEO in complex food matrices and its interaction mechanisms with food components to facilitate its practical application in food preservation and safety.

## Data Availability

The original contributions presented in this study are included in the article/supplementary material, further inquiries can be directed to the corresponding author.
